# Biostimulatory Action of Vegetal Protein Hydrolysate Compensates for Reduced Strength Nutrient Supply in a Floating Raft System by Enhancing Performance and Qualitative Features of “Genovese” Basil

**DOI:** 10.3389/fpls.2022.906686

**Published:** 2022-05-23

**Authors:** Michele Ciriello, Luigi Formisano, Marios C. Kyriacou, Giuseppe Colla, Giulia Graziani, Alberto Ritieni, Stefania De Pascale, Youssef Rouphael

**Affiliations:** ^1^Department of Agricultural Sciences, University of Naples Federico II, Naples, Italy; ^2^Department of Vegetable Crops, Agricultural Research Institute, Aglantzia, Cyprus; ^3^Department of Agriculture and Forest Sciences, University of Tuscia, Viterbo, Italy; ^4^Department of Pharmacy, University of Naples Federico II, Naples, Italy

**Keywords:** *Ocimum basilicum* L., biostimulants, hydroponic, nutrient solution concentration, volatiles, phenolics, antioxidant activities, UHPLC/HRMS

## Abstract

The floating raft constitutes a valuable system for growing herbs as it effectuates high yield and prime functional quality. However, the pressing need for advancing sustainability in food production dictates the reduction of chemical fertilizer inputs in such intensive production schemes through innovative cultivation practices. In this perspective, our work appraised the productive and qualitative responses of two “Genovese” basil genotypes (Eleonora and Italiano Classico) grown in a floating raft system with nutrient solutions of varied electrical conductivity (EC; 2 and 1 dS m^−1^) combined with root application of protein hydrolysate biostimulant at two dosages (0.15 and 0.3 0 ml L^−1^ of Trainer^®^). The phenolic composition, aromatic profile, and antioxidant activities (ABTS, DPPH, and FRAP) of basil were determined by UHPLC/HRMS, GC/MS, and spectrophotometry, respectively. “Eleonora” demonstrated higher number of leaves (37.04 leaves per plant), higher fresh yield (6576.81 g m^−2^), but lower polyphenol concentration (1440.81 μg g^−1^ dry weight) compared to “Italiano Classico.” The lower EC solution (1 dS m^−1^) increased total phenols (+32.5%), ABTS, DPPH, and FRAP antioxidant activities by 33.2, 17.1, and 15.8%, respectively, and decreased linalool relative abundance by 5.5%. Biostimulant application improved crop performance and increased total phenolic concentration in both genotypes, with the highest phenolic concentration (1767.96 μg g^−1^ dry weight) registered at the lowest dose. Significant response in terms of aromatic profile was detected only in “Eleonora.” Our results demonstrate that the application of protein hydrolysate may compensate for reduced strength nutrient solution by enhancing yield and functional quality attributes of “Genovese” basil for pesto.

## Introduction

The ongoing quest for a healthy lifestyle and modern-day awareness exemplified in “*we are what we eat*” usher consumers to dietary schemes characterized by regular consumption of fruits and vegetables. The association between high consumption of healthy foods and low incidence of chronic disorders is attributed to the beneficial effects of the phytochemical antioxidants typical of plants (Teklić et al., 2021). Phytochemicals are classified into three groups according to their metabolic pathway: phenylpropanoids, alkaloids, and terpenoids (El-Nakhel et al., 2019). Better known as secondary metabolites, these biomolecules, crucial in defense and functional environment-plant interaction (Verma and Shukla, 2015), have always been a natural and indispensable resource for cosmetic, pharmaceutical, and agri-food industries (Mahajan et al., 2020; Ciriello et al., 2021b). Minor plant species, such as herbs, due to a heterogeneous and not fully explored reservoir of secondary metabolites, have rekindled the interest of both consumers and academics (Kwon et al., 2020; Alexopoulos et al., 2021). Basil (*Ocimum basilicum* L., *Lamiaceae*) is an irreplaceable ingredient for traditional Italian dishes (“pesto” and pizza “Margherita”) and the pharma-cosmetic sector (Barátová et al., 2015; Ciriello et al., 2021a,b) due to the biosynthesis of low molecular weight organic compounds (i.e., monoterpenes and phenylpropanoids), responsible for its distinctive aroma (Dias et al., 2016). On the other hand, the outstanding nutraceutical value of basil is mainly attributable to a heterogeneous phenolic profile (rosmarinic acid, chicoric acid, caffeic acid, and p-coumaric acid) that, like aroma, is strongly affected by the interaction between genotype and environment (Jakovljević et al., 2019; Ciriello et al., 2021b). To date, phenolic compounds have become among the most investigated natural molecules (Dias et al., 2016). In addition to having a considerable impact on quality attributes (flavor and color), they possess antioxidant, antifungal, and antimicrobial properties, such as being considered multitarget drugs with potential applications in the agri-food sector as surrogates for artificial preservatives (Filip, 2017). Increasingly extreme environmental conditions combined with the demand for high-quality agricultural production have led the growers to alternative cropping systems (Alexopoulos et al., 2021; Ciriello et al., 2021a; Teklić et al., 2021). In this context, the soilless growing system is a viable strategy for the conversion and redevelopment of abandoned urban and peri-urban areas to full-scale green farms (Wortman, 2015). Among hydroponic systems, the floating system is undoubtedly the one that best lends itself to the production of aromatic herbs such as basil (Ciriello et al., 2020, 2021a). This growing system, in addition to being more economically sustainable (lower production and set-up costs), would guarantee, in line with today’s market demands, a higher production all year round with standardized characteristics (Bonasia et al., 2017; Prinsi et al., 2020; Aktsoglou et al., 2021; Žlabur et al., 2021). In addition, the potential to control and manipulate the composition of the nutrient solution (NS) would positively change secondary metabolism by enhancing the phytochemical properties of grown horticultural products (Scuderi et al., 2011; Maggini et al., 2014; El-Nakhel et al., 2019; Prinsi et al., 2020; Aktsoglou et al., 2021). The unclear effects of dilute NS (nutrient stresses) on leafy vegetable yield parameters (Fallovo et al., 2009; El-Nakhel et al., 2019; Ciriello et al., 2020; Hosseini et al., 2021) have prompted growers to use concentrated NS that exceed crop needs (Yang and Kim, 2020). Consistent with the guidelines of the European Commission, the need to reduce chemical input while improving yield and quality in intensive production environments has prompted the agricultural sector to become increasingly interested in biostimulants (Carillo et al., 2019; Kerchev et al., 2020; Teklić et al., 2021). The combination of hydroponic and biostimulants appears to be a promising ecological strategy for controlled environment production of high-quality vegetables. Colla et al. (2015) reported that plant protein hydrolysates (PH’s) are innovative strategies to address the above challenges. Recent work by Rouphael et al. (2021) pointed out that the use of PH’s by foliar application improved the production performance of “Genovese” basil in protected cultures. The effectiveness of these natural products (derived from agricultural by-products) is also confirmed in the work of Caruso et al. (2019) and Cristofano et al. (2021) on arugula (*Eruca sativa* Mill.) and lettuce (*Lactuca sativa* L.), respectively. These results are attributable to bioactive molecules (amino acids and signaling peptides) that exert a plethora of physiological and growth effects on plants while inducing up-regulation of increasingly sought-after secondary metabolites (Colla et al., 2017; Ertani et al., 2017; Caruso et al., 2019). The possibility of sustainably increasing resource use efficiency (Rouphael et al., 2021) by partially ameliorating environmental drawbacks associated with overfertilization makes the application of PH’s in floating raft systems (FRS) even more attractive. The complete absence of interactions between the roots and the agricultural soil makes the FRS suitable for studying *in vivo* the real plant responses to biostimulant integration (Tsouvaltzis et al., 2020). To date, there is a lack of information in the literature on the application mode recommended for this cropping system. However, considering that PH’s improve the uptake, assimilation, and translocation of nutrients through modifications of the root system, the possibility of applying the biostimulant directly in contact with the root system (in NS) could further enhance its potential. The benefits of biostimulus on production and quality in a soilless superintensive system could be an effective tool to reduce chemical fertilizer inputs, and to improve economic and environmental sustainability. Our work aimed to evaluate the use of a PH in a NS at two different doses to assess the effects on the production performance and quality of two Genovese basil genotypes (Eleonora and Italiano Classico) grown in a FRS with two different nutrient concentrations (1 and 2 dS m^−1^).

## Materials and Methods

### Plant Material, Experimental Design, and Growth Conditions

The experimental trial was carried out in a passive ventilation greenhouse at the experimental site of the Federico II University of Naples - Department of Agriculture (DIA) located in Portici (Naples, Italy; lat. 40°51’N, long. 14°34′E; 60 m above sea level), during the summer growing season in 2020. The experimental design was trifactorial in which two “Genovese” basil (*Ocimum basilicum* L.) genotypes [(1) Eleonora, Enza Zaden, Enkhuizen, Noord-Holland, The Netherlands: erect stem, large green, slightly serrated leaves; suitable for open field cultivation; intermediate resistance to *Peronospora belbahrii* and (2) Italiano Classico, La Semiorto Sementi, Sarno, SA, Italy; erect stem, medium height with bright green, slightly blistered “spoon” leaves] were grown in a FRS with two different concentrations of NS (1 dS m^−1^-Half Strength and 2 dS m^−1^-Full Strength) and two doses of biostimulants (0.15 and 0.30 ml L^−1^) plus an untreated control (hereafter B_0.15_, B_0.30_, and Control, respectively). The treatments were performed in triplicate and arranged in a completely randomized block design. On 9 June (18 days after sowing), at the phenological stage of 2–3 true leaves, basil seedlings were transplanted into 54-hole polystyrene trays (52 × 32 × 6 cm; upper hole diameter: 4.5 cm; bottom hole diameter 3 cm; volume: 0.06 l) at a density of 317 plants m^−2^. The experimental design comprised 36 experimental units, each consisting of a 54-hole tray floating in a 40-liter tank filled with 35 l of NS. The oxygenation of the NS was provided by a submersible pump (Aquaball 60, Eheim, Stuttgart, Germany).

### Nutrient Solutions Management, Biostimulant Application, and Harvest

The NS (half strength and full strength) were prepared from osmosis water. The half strength NS was obtained by halving the macronutrient concentration of the full strength stock NS (14.0 mM nitrate, 1.5 mM phosphorus, 3.0 mM potassium, 1.75 mM sulfur, 4.5 mM calcium, 1.5 mM magnesium, and 1.0 mM ammonium). Micronutrient concentrations were for both solutions 15 μM iron, 9 μM manganese, 0.3 μM copper, 1.6 μM zinc, 20 μM boron, and 0.3 μM molybdenum. During the trial, the pH was continuously monitored and maintained at values of 5.8 ± 0.2. At transplanting, a legume PH (Trainer- Hello Nature USA Inc., Anderson, IN 46016) was applied to the NS at two different doses (0.15 ml L^−1^ and 0.30 ml L^−1^). The biostimulant used, which was free of plant hormones (Rouphael et al., 2018; Paul et al., 2019), contained soluble peptides and amino acids such as Ala, Arg, Asp., Cys, Glu, Gly, His, Ile, Leu, Lys, Met, Phe, Pro, Ser, Thr, Trp, Tyr, and Val, which comprised 5% of the total nitrogen content along with soluble sugars and phenols. At the end of the experiment, 25 plants per experimental unit were sampled to determine biometric parameters such as the number of leaves per plant and fresh yield. The harvested plant material was then placed in a ventilated oven at 60°C until a constant weight was reached to determine the dry yield and the percentage of dry matter (DM = 100 × dry weight/fresh weight). Instead, a homogeneous pool of 20 plants per experimental unit was sampled and placed immediately at −80°C for future qualitative analysis. A plant material sample was freeze-dried (Alpha 1–4, Martin Christ Gefriertrocknungsanlagen GmbH, Osterode am Harz, Germany) and finely ground with a KM13 rotating blade grinder (Bosch, Gerlingen, Germany).

### CIELab Color Space Determination

At harvest, color coordinates were recorded on the adaxial surface of ten healthy and fully expanded leaves per experimental unit using a Minolta Chromameter CR-400 portable colorimeter (Minolta Camera Co. Ltd., Osaka, Japan). As described by the International Commission on Illumination (CIE), the color was expressed by L, a^*^, and b^*^ coordinates by which the Chroma and Hue angle were determined as follows:


$$\mathrm{Chroma}={\left[{\left(a^{*}\right)}^{2}+{\left(b^{*}\right)}^{2}\right]}^{0.5}$$

$$\mathrm{Hue}\;\mathrm{angle}=\tan^{-1}\;b^{*}/a^{*}$$

### Determination of ABTS, DPPH, and FRAP Antioxidant Activities

The antioxidants activities were determined following the protocols described by Graziani et al. (2021). For the determination of ABTS antioxidant activity a stock solution was prepared by mixing 44 ml of potassium persulfate (2.45 mM) with 2.50 ml of aqueous solution (7 mM) of 2,2′-azinobis-(3-ethylbenzothiazoline-6-sulfonate) radical (ABTS^+^) and placed at 20°C (room temperature) for 12 h. The ABTS solution was diluted (1:88) with ethanol until it reached an absorbance of 0.700 ± 0.005 at 734 nm. After that, a 1 ml aliquot of ABTS solution was added to 100 ml of the filtered sample and incubated at room temperature for 2.5 min. For the antioxidant 2,2-diphenyl-1-picryl-hydrazyl (DPPH) activity, 1 ml of methanolic solution of DPPH 100 μM (absorbance of 0.90 ± 0.02 at 517 nm) was added to 0.2 ml of diluted leaf extract and incubated at room temperature for 10 min. For ferric reduction/antioxidant power (FRAP) antioxidant activity determination a FRAP reagent was prepared by mixing 1.25 ml of 2,4,6-triridyl-striazine (TPTZ; 10 mM) in 40 mM Hydrochloric acid, 1.25 ml of 20 mmol ferric chloride in water, and 12.5 ml of 0.3 M sodium acetate (pH 3.6). An aliquot of 2.850 ml of FRAP reagent was added to 0.015 ml of leaf extract and incubated at room temperature for 4 min. The absorbances of the ABTS, DPPH, and FRAP assays were measured with a UV–VIS spectrophotometer (Shimadzu, Japan) at 734, 517, and 593 nm, respectively. Results were expressed as mmol Trolox equivalents kg^−1^ dry weight (dw) of the sample. All determinations were made in triplicate.

### Determination of the Polyphenol Profile by Ultra-High Performance Liquid Chromatography and Orbitrap High-Resolution Mass Spectrometry Analysis

#### Extraction of Polyphenolic Compounds

Polyphenolic compounds were extracted as described by Corrado et al. (2021). Briefly, 0.1 g of finely ground and freeze-dried leaves was extracted in 5 ml of an aqueous methanol solution (60:40, *v/v*). Then, the obtained solution was sonicated and centrifuged at 4,000 rpm for 15 min, and 0.05 ml of supernatant was collected, filtered, and analyzed.

#### Quantification of Phenolic Compounds

Quantification and separation of phenolic compounds were performed by UltraHigh-Pressure Liquid Chromatography (Dionex UltiMate 3,000 UHPLC, Thermo Fisher Scientific, Waltham, MA, United States) coupled to the Q Exactive Orbitrap LC–MS/MS Mass Spectrometer (Thermo Fisher Scientific, Waltham, MA, United States) as described by El-Nakhel et al. (2021). The polyphenols were separated by using a Luna Omega PS (1.6 m, 50 × 2.1 mm, Phenomenex, Torrance, CA, United States) at 25 ° C. The mobile phase was a two-phase solution containing water (phase A) and acetonitrile (phase B). Both mobile phases contained 0.1% formic acid (*v/v*). Polyphenolic compounds were eluted using the following gradient schedule: 0–1.3 min 5% B, 1.3–9.3 min 5–100% B, 9.3–11.3 min 100% B, 11.3–13.3 min 100–5% B, 13.3–20 min 5% B. The flow rate was 0.2 ml min^−1^. For all compounds of interest, an ESI source (Thermo Fisher Scientific, Waltham, MA, United States) was used in negative ion mode, with full ion (MS) and all ion fragmentation (AIF) scanning events. Data acquisition and processing were performed with Quan/Qual Browser Xcalibur software, v. 3.1.66.10 Xcalibur, Thermo Fisher Scientific, (Thermo Fisher Scientific, Waltham, MA, United States). Polyphenols were expressed as μg g^−1^ dw.

### Determination of Volatile Compounds

The extraction and quantification of volatile compounds (VOCs) were performed by gas chromatography combined with the mass spectrometer technique (GC/MS) after solid phase microextraction (SPME), as described in detail by Ciriello et al. (2021b).

#### Extraction of Volatile Compounds by the SPME Technique

An aliquot of 0.5 g of frozen sample was placed in glass vials with a screw cap and placed on a heated stirrer (30°C for 10 min) to facilitate the migration of volatile compounds into the headspace. The adsorption of VOCs was performed by introducing a divinylbenzene/carboxane/polydimethylsiloxane fiber 1 cm long and 50/30 μm thick; Supelco^®^ (Bellefonte, PA, United States) into the headspace for 10 min.

#### Quantification of Volatile Compounds

SPME fiber containing the adsorbed analytes was introduced into the split–splitless injector of the gas chromatograph (GC 6890 N; Agilent, Santa Clara, CA, United States) coupled to the mass spectrometer (MS 5973 N; Agilent, Santa Clara, California, United States). The thermal desorption of the analytes occurred at 250 ° C for 10 min. The oven temperature was maintained at 50°C for 2 min and increased from 50°C to 150°C at 10°C/min and from 150°C to 280°C at 15°C/min. The injection and ion source temperatures were 250°C and 230°C, respectively, and helium (99.999%) was used as a carrier gas with a flow rate of 1 ml min^−1^. The gas chromatograph was equipped with a capillary column (30 m × 0.250 mm) coated with a 0.25 μm 5% diphenyl/95% dimethylpolysiloxane film (Supelco^®^, Bellefonte, PA, United States). The mass spectrometer was set at 70 eV. Identification of VOCs identification was performed using the National Institute of Standards and Technology (NIST) Atomic Spectra Database version 1.6 (U.S. Department of Commerce, Gaithersburg, Maryland, United States).

### Statistics

The experiment consisted of a randomized block design with three factors: Cultivar-CV, Biostimulant-B, and Nutrient Solution Concentration-NSC. Analysis of variance (ANOVA) was conducted for the main effects and their interactions. In the absence of significant interactions, significant main effects for factors applied at only two levels (CV and NSC) also denote significant differences between the two means. In the case of significant two-way interactions (CV × B, B × NSC, and CV × NSC), interaction means were compared using the Tukey–Kramer HSD test with statistical significance determined at the *p* < 0.05 level. All data are presented as mean ± standard error. Statistical analysis was performed using IBM SPSS 20 (Armonk, NY, United States) package for Microsoft Windows 10. Statistical processing was performed using IBM SPSS 20 (Armonk, NY, United States) package for Microsoft Windows 10.

## Results

### Yield and Yield Parameters

Regarding the main yield parameters, the cultivar factor significantly influenced all the parameters reported in Table 1, except the dry yield. Although “Eleonora” had the highest number of leaves and the highest fresh yield, “Italiano Classico” was characterized by a higher percentage of dry matter. Biostimulant treatment significantly influenced all yield parameters compared to the Nutrient Solution Concentration that affected dry yield and dry matter (Table 1).

**Table 1:** Analysis of variance and mean comparisons for leaf number, fresh yield, dry yield, and dry matter of Eleonora and Italiano Classico genotypes grown hydroponically under two nutrient solution and dose of biostimulant.

**Treatment**	Leaf number	Fresh Yield	Dry Yield	Dry matter
	No. plant^−1^	(g m^−2^)	(g m^−2^)	(%)
**Cultivar (CV)**				
Eleonora	37.04 ± 0.35	6576.81 ± 126.42	486.40 ± 10.47	7.40 ± 0.06
Italiano Classico	31.34 ± 0.64	6232.70 ± 124.58	481.99 ± 11.89	7.76 ± 0.05
**Biostimulant (B)**				
Control	31.86 ± 1.15c	5863.99 ± 69.31c	434.65 ± 5.43c	7.40 ± 0.06c
B_0.15_	34.49 ± 0.88b	6365.64 ± 99.00b	490.34 ± 6.12b	7.78 ± 0.05a
B_0.30_	36.21 ± 0.71a	6984.64 ± 95.94a	527.60 ± 10.88a	7.57 ± 0.10b
**Nutrient Solution Concentration (NSC)**				
Half Strength (HS)	34.33 ± 0.82	6406.93 ± 97.33	479.18 ± 7.47	7.48 ± 0.05
Full Strength (FS)	34.05 ± 0.91	6402.59 ± 159.72	489.21 ± 13.88	7.68 ± 0.08
**CV × B**				
Eleonora × Control	35.46 ± 0.44bc	6016.81 ± 102.01	435.60 ± 9.8d	7.22 ± 0.03d
Eleonora × B_0.15_	37.23 ± 0.28ab	6601.58 ± 120.67	503.25 ± 6.46bc	7.66 ± 0.08bc
Eleonora × B_0.30_	38.43 ± 0.32a	7112.04 ± 133.79	520.34 ± 13.32ab	7.32 ± 0.06d
Italiano Classico × Control	28.27 ± 0.68f	5711.17 ± 37.28	433.70 ± 5.77d	7.58 ± 0.05c
Italiano Classico × B_0.15_	31.74 ± 0.55d	6129.71 ± 79.32	477.42 ± 7.49c	7.89 ± 0.04a
Italiano Classico × B_0.30_	34.00 ± 0.35c	6857.24 ± 126.91	534.86 ± 17.95a	7.81 ± 0.11ab
**B × NSC**				
Control × HS	32.40 ± 1.82	5979.71 ± 119.42 cd	442.53 ± 7.57c	7.38 ± 0.06c
B_0.15_ × HS	34.58 ± 0.99	6525.28 ± 136.88b	500.34 ± 8.59b	7.67 ± 0.07b
B_0.30_ × HS	36.01 ± 1.11	6715.79 ± 72.40b	494.67 ± 5.69b	7.40 ± 0.09c
Control × FS	31.33 ± 1.55	5748.27 ± 38.99d	426.76 ± 6.88c	7.42 ± 0.12c
B_0.15_ × FS	34.39 ± 1.56	6206.01 ± 119.12c	480.33 ± 7.13b	7.88 ± 0.06a
B_0.30_ × FS	36.42 ± 0.96	7253.49 ± 79.66a	560.53 ± 7.39a	7.74 ± 0.14ab
**CV × NSC**				
Eleonora × HS	37.15 ± 0.36	6624.76 ± 102.91	487.73 ± 9.05ab	7.34 ± 0.05d
Eleonora × FS	36.92 ± 0.63	6528.87 ± 238.23	485.06 ± 19.59ab	7.46 ± 0.10c
Italiano Classico × HS	31.50 ± 0.85	6189.10 ± 133.44	470.63 ± 11.72b	7.63 ± 0.05b
Italiano Classico × FS	31.17 ± 1.01	6276.31 ± 218.37	493.35 ± 20.76a	7.89 ± 0.06a
**Significance**				
**CV**	^***^	^***^	ns	^***^
**B**	^***^	^***^	^***^	^***^
**NSC**	ns	ns	^*^	^***^
**CV× B**	^**^	ns	^**^	^**^
**B × NSC**	ns	^***^	^***^	^**^
**CV × NSC**	ns	ns	^*^	^*^

Nutrient solution concentration treatments: HS, half strength; FS, full strength; Biostimulant treatments: Control; B_0.15_ = 0.15 ml L^−1^ of Trainer^®^; B_0.30_ = ml L^−1^ of Trainer^®^. ns, non-significant effect. Data represent means ± standard error of 3 replicates (*n* = 3). Treatment means within each column followed by different letters denote significant differences (*p* < 0.05) according to Tukey–Kramer HSD test.

* Significant effect at the 0.05 level.

** Significant effect at the 0.01 level.

*** Significant effect at the 0.001 level.

**Figure 1:**
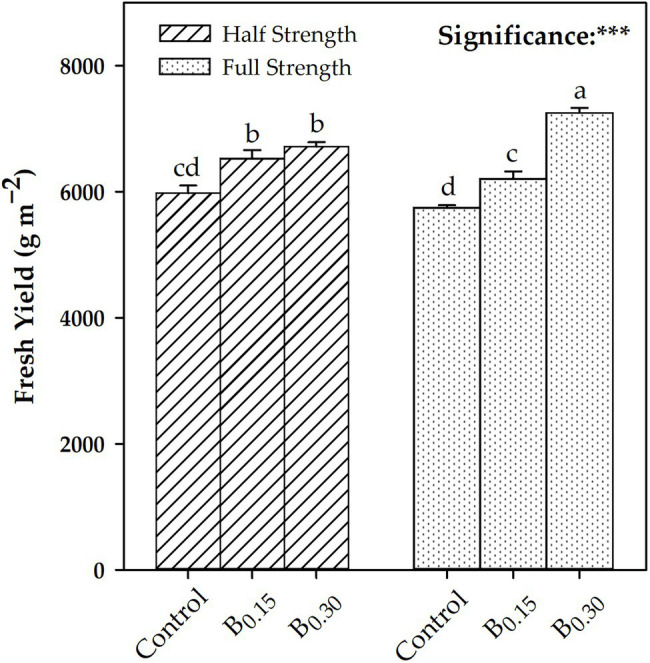
Effects of Biostimulant × Nutrient Solution Concentration interaction for fresh yield [Nutrient solution concentration treatments: Half strength and Full Strength; biostimulant treatments: Control; B0.15 = 0.15 ml L^−1^ of Trainer^®^; B0.30 = ml L^−1^ of Trainer^®^]. Different letters denote significant differences (*p* < 0.05) according to Tukey–Kramer HSD test. ***Significant effect at the 0.001 level.

Biostimulant treatment showed a linear increase in leaf number, fresh yield, and dry yield as a function of the dose used (Control > B_0.15_ > B_0.30_), in contrast to dry matter, which showed the highest value at B_0.15_. Regarding the CV × B interaction for both “Eleonora” and “Italiano Classico,” the B_0.30_ dose determined, compared to the Control, an average increase of 13.65 and 21.38% in the number of leaves and dry yield, respectively. The dry matter did not show the same trend since, in “Eleonora,” the highest value was obtained at B_0.15_, while in “Italiano Classico,” the highest values were obtained at B_0.15_ and B_0.30_. The CV × B interaction did not influence fresh yield that was significantly influenced by the B × NSC interaction (Figure 1). Regardless of the NSC, the use of the biostimulant increase fresh yield (Figure 1) and dry yield (Table 1). However, for the full strength solution (FS; 2 dS m^−1^), a linear increase of the above two parameters was observed as the concentration of the biostimulant increased. The highest dry matter values were obtained at B_0.15_ for both half strength [HS;1 dS m^−1^; (7.67%)] and FS (7.88%) nutrient solutions. On the contrary, the highest values were already observed at B_0.15_ for the HS nutrient solution, which did not show significant differences compared to the B_0.30_ dose. The CV × NSC interaction showed significant differences only for dry yield and dry matter. In “Italiano Classico,” the use of the FS increases by 4.82 and 3.40% dry yield and dry matter, respectively. The same trend was observed in “Eleonora” only for dry matter (+1.60%).

### CIELab Colorimetric Parameters

Except for the Hue angle, significant differences were observed between genotypes for leaf colorimetric characteristics (Table 2). “Italiano Classico” showed the highest values of L, a^*^, b^*^, and Chroma. Greenness (a^*^) was the only parameter influenced by the biostimulant, with the highest value obtained at B_0.30_. The different NSC influenced the colorimetric parameters L, a^*^, and Hue angle, as opposed to b^*^ and Chroma. The latter were significantly affected by the CV × B and CV × NSC interactions (Table 2). The CV × NSC interaction also influenced the a^*^ and Hue angle parameters. The biostimulants in the HS solutions did not affect a^*^ and Hue angle, compared to the FS nutrient solution, where the B_0.15_ and B_0.30_ doses increased these parameters, compared to the Control. The CV × NSC interaction showed significant differences only for the parameters L and b^*^. In both “Eleonora” and “Italiano Classico,” the use of HS increased L; the opposite trend was observed for the b^*^ in Eleonora, while no significant differences were registered in “Italiano Classico.”

**Table 2:** Analysis of variance and mean comparisons for CIELab colorimetric parameters of Eleonora and Italiano Classico genotypes grown hydroponically under two nutrient solution and dose of biostimulant.

**Treatment**	L^*^	a^*^	b^*^	**Chroma**	**Hue angle**
**Cultivar (CV)**					
Eleonora	44.99 ± 0.15	−6.92 ± 0.14	15.97 ± 0.17	17.04 ± 0.16	112.69 ± 0.11
Italiano Classico	45.36 ± 0.10	−7.17 ± 0.12	16.54 ± 0.12	18.09 ± 0.18	112.52 ± 0.18
**Biostimulant (B)**					
Control	45.15 ± 0.17	−6.82 ± 0.06b	16.38 ± 0.19	17.83 ± 0.26	112.51 ± 0.14
B_0.15_	45.32 ± 0.14	−7.04 ± 0.21ab	16.35 ± 0.23	17.40 ± 0.17	112.61 ± 0.21
B_0.30_	45.06 ± 0.18	−7.28 ± 0.15a	16.04 ± 0.16	17.47 ± 0.33	112.70 ± 0.19
**Nutrient Solution Concentration (NSC)**					
Half Strength (HS)	45.57 ± 0.06	−6.73 ± 0.08	16.17 ± 0.16	17.45 ± 0.18	112.41 ± 0.15
Full Strength (FS)	44.78 ± 0.11	−7.36 ± 0.13	16.34 ± 0.16	17.68 ± 0.24	112.80 ± 0.13
**CV × B**					
Eleonora × Control	44.93 ± 0.28	−6.72 ± 0.08	15.90 ± 0.16bc	17.39 ± 0.18c	112.53 ± 0.14
Eleonora × B_0.15_	45.18 ± 0.26	−6.92 ± 0.33	16.33 ± 0.45ab	17.21 ± 0.31 cd	112.57 ± 0.24
Eleonora × B_0.30_	44.86 ± 0.25	−7.13 ± 0.25	15.67 ± 0.14c	16.51 ± 0.24d	112.96 ± 0.11
Italiano Classico × Control	45.37 ± 0.16	−6.91 ± 0.07	16.85 ± 0.22a	18.27 ± 0.44ab	112.49 ± 0.26
Italiano Classico × B_0.15_	45.46 ± 0.09	−7.16 ± 0.29	16.37 ± 0.18ab	17.58 ± 0.10bc	112.65 ± 0.36
Italiano Classico × B_0.30_	45.27 ± 0.24	−7.43 ± 0.17	16.41 ± 0.19ab	18.43 ± 0.22a	112.43 ± 0.35
**B × NSC**					
Control × HS	45.56 ± 0.14	−6.88 ± 0.08b	16.61 ± 0.23ab	17.61 ± 0.19ab	112.84 ± 0.14ab
B_0.15_ × HS	45.59 ± 0.09	−6.41 ± 0.08b	15.74 ± 0.18c	16.99 ± 0.18b	112.05 ± 0.19c
B_0.30_ × HS	45.57 ± 0.12	−6.91 ± 0.15b	16.17 ± 0.31bc	17.76 ± 0.46ab	112.32 ± 0.31bc
Control × FS	44.73 ± 0.19	−6.75 ± 0.08b	16.14 ± 0.30bc	18.04 ± 0.50a	112.18 ± 0.16c
B_0.15_ × FS	45.06 ± 0.21	−7.67 ± 0.18a	16.96 ± 0.23a	17.81 ± 0.14ab	113.16 ± 0.16a
B_0.30_ × FS	44.56 ± 0.15	−7.65 ± 0.15a	15.91 ± 0.10c	17.18 ± 0.48ab	113.07 ± 0.09a
**CV × NSC**					
Eleonora × HS	45.53 ± 0.10a	−6.54 ± 0.09	15.71 ± 0.15b	17.06 ± 0.21	112.61 ± 0.17
Eleonora × FS	44.46 ± 0.10c	−7.30 ± 0.19	16.23 ± 0.28a	17.01 ± 0.27	112.76 ± 0.13
Italiano Classico × HS	45.62 ± 0.08a	−6.92 ± 0.11	16.64 ± 0.17a	17.85 ± 0.25	112.21 ± 0.23
Italiano Classico × FS	45.11 ± 0.13b	−7.41 ± 0.19	16.45 ± 0.17a	18.34 ± 0.25	112.84 ± 0.24
**Significance**					
**CV**	^*^ ^*^ ^*^	^*^	^***^	^*^^**^	ns
**B**	ns	^**^	ns	ns	ns
**NSC**	^***^	^***^	ns	ns	^**^
**CV× B**	ns	ns	^*^	^***^	ns
**B × NSC**	ns	^***^	^**^ ^*^	^**^	^***^
**CV × NSC**	^**^	ns	^**^	ns	ns

Nutrient solution concentration treatments: HS, half strength; FS, full strength; Biostimulant treatments: Control; B_0.15_ = 0.15 ml L^−1^ of Trainer^®^; B_0.30_ = ml L^−1^ of Trainer^®.^ ns = non-significant effect. Data represent means ± standard error of 3 replicates (*n* = 3). Treatment means within each column followed by different letters denote significant differences (*p* < 0.05) according to Tukey–Kramer HSD test.

* Significant effect at the 0.05 level.

** Significant effect at the 0.01 level.

*** Significant effect at the 0.001 level.

### Phenolic Acids

The total phenols were influenced by the factors under investigation and their mutual interactions (Table 3). Chicoric acid was the predominant compound, followed by feruloyl tartaric acid, salvianolic acid K, rosmarinic acid, caftaric acid, salvianolic acid L, and chlorogenic acid. “Italiano Classico” showed the highest content of chicoric acid, salvianolic acid K, rosmarinic acid, salvianolic acid L, and chlorogenic acid, while “Eleonora” showed the highest concentration of feruloyl tartaric acid. B and NSC treatments significantly affected the entire phenolic profile (Table 3). Specifically, the biostimulant at B_0.15_ dose increased the total phenols by 35.63% compared to the Control. Similarly, the HS increased the total phenol by 32.50%, compared to the HS one. CV × B interaction affected all the parameters reported in Table 3. The B_0.15_ dose increased caftaric acid, feruloyl tartaric acid, salvianolic acid K, salvianolic acid L, and total phenols for both genotypes, compared to the Control. On the other hand, the highest chicoric acid values were obtained from the Italiano Classico × B_0.15_ combination (1290.32 μg g^−1^ dw) and Italiano Classico × B_0.30_ (1309.98 μg g^−1^ dw). In comparison, the lowest value was obtained from the combination Eleonora × B_0.30_ (11.68 μg g^−1^ dw). Except for the most and least representative phenolic acids (chicoric and chlorogenic acids, respectively), all phenolic acids were affected by the B × NSC interaction. The B_0.15_ × HS combination provided the highest total phenol concentration (2045.94 μg g^−1^ dw) and feruloyl tartaric acid, salvianolic acid L, salvianolic acid K, and caftaric acid, while the highest concentration of rosmarinic acid was obtained from the Control × HS combination. Compared to the CV × NSC interaction, for both “Eleonora” and “Italiano Classico,” the HS, compared to the FS, increased total phenols by 53.99 and 15.95%, respectively. Except for caftaric acid, the highest concentration of all phenolic acids was recorded for both genotypes in the HS.

**Table 3:** Analysis of variance and mean comparisons for phenolic acids in Eleonora and Italiano Classico genotypes grown hydroponically under two nutrient solution and dose of biostimulant.

**Treatment**	**Caftaric acid**	**Chlorogenic acid**	**Feruloyl tartaric acid**	**Salvianolic acid K**	**Salvianolic acid L**	**Rosmarinic acid**	**Cichoric acid**	**Total Phenols**
**Cultivar (CV)**								
Eleonora	48.41 ± 4.78	40.29 ± 1.36	243.49 ± 9.56	42.27 ± 8.03	39.52 ± 9.65	36.28 ± 6.21	990.57 ± 59.63	1440.81 ± 92.40
Italiano Classico	49.30 ± 5.66	42.84 ± 2.01	166.68 ± 13.6	71.62 ± 5.66	50.96 ± 7.56	61.45 ± 3.08	1147.18 ± 59.55	1590.04 ± 86.65
**Biostimulant (B)**
Control	32.74 ± 6.48c	35.38 ± 1.39c	166.50 ± 18.61c	45.01 ± 8.31c	28.99 ± 4.75b	57.78 ± 5.52a	937.10 ± 49.81c	1303.50 ± 80.99c
B_0.15_	63.45 ± 5.05a	46.04 ± 1.85a	250.84 ± 13.19a	74.86 ± 10.64a	77.73 ± 13.62a	49.12 ± 5.80b	1205.92 ± 71.14a	1767.96 ± 111.20a
B_0.30_	50.38 ± 4.10b	43.27 ± 1.77b	197.92 ± 13.86b	50.97 ± 7.43b	29.00 ± 2.32b	39.69 ± 8.69c	1063.60 ± 85.47b	1474.83 ± 98.81b
**Nutrient Solution Concentration (NSC)**
Half Strength (HS)	58.34 ± 4.32	45.45 ± 1.33	232.89 ± 15.13	78.49 ± 6.65	65.08 ± 9.21	60.38 ± 5.75	1186.69 ± 39.37	1727.32 ± 65.05
Full Strength (FS)	39.37 ± 5.07	37.68 ± 1.59	177.28 ± 11.40	35.40 ± 4.82	25.40 ± 4.81	37.35 ± 4.24	951.05 ± 68.12	1303.54 ± 84.79
**CV × B**								
Eleonora × Control	47.08 ± 9.54c	38.36 ± 1.61d	223.51 ± 12.48c	28.72 ± 4.59d	27.56 ± 9.44c	62.87 ± 10.17b	1032.96 ± 38.36b	1461.07 ± 83.74d
Eleonora × B_0.15_	56.88 ± 9.52b	42.28 ± 3.02c	269.12 ± 20.79a	66.02 ± 20.85bc	65.8 ± 25.4b	34.28 ± 4.61d	1121.53 ± 133.94b	1655.90 ± 216.47c
Eleonora × B_0.30_	41.27 ± 5.16d	40.22 ± 2.38 cd	237.85 ± 11.21b	32.06 ± 5.65d	25.19 ± 2.90c	11.68 ± 2.24e	817.21 ± 84.00c	1205.47 ± 113.17e
Italiano Classico × Control	18.40 ± 3.35e	32.41 ± 1.55e	109.49 ± 8.22e	61.30 ± 13.28c	30.41 ± 3.09c	52.68 ± 4.47c	841.24 ± 75.96c	1145.93 ± 109.18e
Italiano Classico × B_0.15_	70.02 ± 2.06a	49.80 ± 0.57a	232.56 ± 14.12bc	83.69 ± 5.65a	89.67 ± 10.67a	63.96 ± 6.24ab	1290.32 ± 38.46a	1880.01 ± 50.23a
Italiano Classico × B_0.30_	59.49 ± 3.76b	46.33 ± 2.1b	158.00 ± 9.03d	69.88 ± 8.24b	32.81 ± 3.09c	67.70 ± 3.67a	1309.98 ± 28.34a	1744.19 ± 33.61b
**B × NSC**								
Control × HS	47.03 ± 9.56c	38.72 ± 1.44	189.49 ± 27.69c	64.88 ± 11.67b	42.57 ± 3.27b	73.92 ± 5.34a	1061.13 ± 26.38	1517.73 ± 58.58c
B_0.15_ × HS	76.00 ± 1.56a	49.57 ± 0.61	289.62 ± 11.66a	104.13 ± 4.63a	117.13 ± 5.68a	61.03 ± 7.53b	1348.48 ± 38.73	2045.94 ± 47.29a
B_0.30_ × HS	51.98 ± 0.47b	48.07 ± 1.35	219.56 ± 19.39b	66.46 ± 9.76b	35.54 ± 1.92bc	46.19 ± 13.22c	1150.48 ± 70.42	1618.28 ± 77.63b
Control × FS	18.45 ± 3.38d	32.04 ± 1.42	143.50 ± 23.35e	25.15 ± 3.07e	15.40 ± 3.86c	41.64 ± 1.09 cd	813.07 ± 63.78	1089.26 ± 84.11e
B_0.15_ × FS	50.89 ± 6.83bc	42.52 ± 3.13	212.06 ± 5.27b	45.59 ± 11.57c	38.34 ± 12.76bc	37.21 ± 5.89de	1063.37 ± 112.42	1489.97 ± 145.81c
B_0.30_ × FS	48.77 ± 8.53bc	38.48 ± 1.67	176.29 ± 16.78d	35.47 ± 7.19d	22.46 ± 1.73bc	33.20 ± 11.86e	976.72 ± 155.44	1331.38 ± 169.42d
**CV × NSC**								
Eleonora × HS	66.37 ± 3.78a	45.35 ± 1.07a	276.41 ± 10.05a	65.26 ± 11.9b	67.16 ± 14.2a	48.77 ± 10.03b	1177.81 ± 63.18ab	1747.12 ± 102.13a
Eleonora × FS	30.45 ± 1.51c	35.22 ± 0.58c	210.57 ± 4.01b	19.27 ± 0.61d	11.88 ± 1.86c	23.79 ± 4.92c	803.33 ± 48.52c	1134.50 ± 49.01c
Italiano Classico × HS	50.30 ± 6.99b	45.55 ± 2.53a	189.37 ± 19.95c	91.71 ± 1.51a	63.00 ± 12.55a	71.99 ± 2.49a	1195.58 ± 50.74a	1707.51 ± 86.35a
Italiano Classico × FS	48.3 ± 9.34b	40.14 ± 2.98b	143.99 ± 16.09d	51.54 ± 5.76c	38.92 ± 7.01b	50.91 ± 2.53b	1098.78 ± 109.14b	1472.58 ± 144.94b
**Significance**								
**CV**	ns	^***^	^***^	^***^	^***^	^***^	^***^	^***^
**B**	^***^	^***^	^***^	^***^	^***^	^***^	^***^	^***^
**NSC**	^***^	^***^	^***^	^***^	^***^	^***^	^***^	^***^
**CV× B**	^***^	^***^	^***^	^***^	^**^	^***^	^***^	^***^
**B × NSC**	^***^	ns	^***^	^***^	^***^	^***^	ns	^***^
**CV × NSC**	^***^	^***^	^***^	^*^	^***^	^*^	^***^	^***^

Nutrient solution concentration treatments: HS, half strength; FS, full strength; Biostimulant treatments: Control; B_0.15_ = 0.15 ml L^−1^ of Trainer^®^; B_0.30_ = ml L^−1^ of Trainer^®^. All data are expressed as μg g^−1^ dw. ns = non-significant effect. Data represent means ± standard error of 3 replicates (*n* = 3). Treatment means within each column followed by different letters denote significant differences (*p* < 0.05) according to Tukey–Kramer HSD.

* Significant effect at the 0.05 level.

** Significant effect at the 0.01 level.

*** Significant effect at the 0.001 level.

### Antioxidant Activities

The results of the ABTS, DPPH, and FRAP assay are presented in Table 4 and are expressed as Trolox equivalents mmol kg^−1^ dw. The CV factor did not result in any significant differences for all antioxidant activities, in contrast to what was observed for the B and NSC factors. Specifically, application of Biostimulant at B_0.15_ dose increased ABTS, DPPH, and FRAP by 32.37, 31.37, and 19.80%, respectively, compared to B_0.30_ dose. Relative to the effect of nutrient solution concentrations, HS resulted in a significant increase in all antioxidant activities compared to FS. The CV × B and B × NSC interactions did not result in significant differences for all parameters reported in Table 4, compared to the CV × NSC interaction where differences were observed only for DPPH antioxidant activity. In “Italiano Classico,” the different nutrient solution concentration did not lead to significant differences for this parameter (DPPH). In contrast, in “Eleonora,” the FS reduced DPPH by 27.32%, compared to the HS.

**Table 4:** Analysis of variance and mean comparisons for ABTS, DPPH, and FRAP antioxidant activities of Eleonora and Italiano Classico genotypes grown hydroponically under two nutrient solution and dose of biostimulant.

**Treatment**	**ABTS**	**DPPH**	**FRAP**
	**(mmol Trolox eq. kg**^−**1**^ **dw)**	**(mmol Trolox eq. kg**^−**1**^ **dw)**	**(mmol Trolox eq. kg**^−**1**^ **dw)**
**Cultivar (CV)**			
Eleonora	39.84 ± 2.87	28.63 ± 1.79	49.59 ± 2.24
Italiano Classico	39.02 ± 2.23	31.15 ± 1.62	51.86 ± 2.11
**Biostimulant (B)**			
Control	40.49 ± 2.69ab	30.30 ± 1.58ab	50.60 ± 2.34ab
B_0.15_	44.32 ± 3.50a	33.71 ± 2.47a	55.36 ± 3.04a
B_0.30_	33.48 ± 2.41b	25.66 ± 1.56b	46.21 ± 1.95b
**Nutrient Solution Concentration (NSC)**			
Half Strength (HS)	45.05 ± 2.42	32.25 ± 1.47	54.44 ± 1.95
Full Strength (FS)	33.81 ± 1.93	27.53 ± 1.80	47.01 ± 2.05
**CV × B**			
Eleonora × Control	42.18 ± 3.95	30.42 ± 1.61	52.34 ± 3.79
Eleonora × B_0.15_	44.65 ± 6.03	31.81 ± 3.98	51.16 ± 4.34
Eleonora × B_0.30_	32.71 ± 4.06	23.66 ± 2.59	45.28 ± 3.50
Italiano Classico × Control	38.81 ± 3.88	30.18 ± 2.89	48.87 ± 2.92
Italiano Classico × B_0.15_	44.00 ± 4.18	35.61 ± 3.09	59.57 ± 3.83
Italiano Classico × B_0.30_	34.24 ± 2.98	27.66 ± 1.54	47.14 ± 2.05
**B × NSC**			
Control × HS	46.84 ± 1.75	31.87 ± 1.37	55.34 ± 2.68
B_0.15_ × HS	52.19 ± 4.23	36.88 ± 2.99	59.04 ± 3.57
B_0.30_ × HS	36.13 ± 3.45	28.01 ± 1.78	48.93 ± 2.91
Control × FS	34.14 ± 3.54	28.72 ± 2.84	45.86 ± 2.81
B_0.15_ × FS	36.46 ± 3.36	30.54 ± 3.73	51.68 ± 4.74
B_0.30_ × FS	30.83 ± 3.29	23.31 ± 2.30	43.49 ± 2.31
**CV × NSC**			
Eleonora × HS	47.85 ± 3.69	33.16 ± 2.34a	55.36 ± 3.22
Eleonora × FS	31.84 ± 2.33	24.10 ± 1.75b	43.82 ± 1.62
Italiano Classico × HS	42.25 ± 3.04	31.34 ± 1.86ab	53.51 ± 2.37
Italiano Classico × FS	35.78 ± 3.06	30.95 ± 2.78ab	50.20 ± 3.55
**Significance**			
**CV**	ns	ns	ns
**B**	^**^	^**^	^*^
**NSC**	^***^	^*^	^**^
**CV× B**	ns	ns	ns
**B × NSC**	ns	ns	ns
**CV × NSC**	ns	^*^	ns

Nutrient solution concentration treatments: HS, half strength; FS, full strength; Biostimulant treatments: Control; B_0.15_ = 0.15 ml L^−1^ of Trainer^®^; B_0.30_ = ml L^−1^ of Trainer^®^ ns, non-significant effect. Data represent means ± standard error of 3 replicates (*n* = 3). Treatment means within each column followed by different letters denote significant differences (*p* < 0.05) according to Tukey–Kramer HSD test.

* Significant effect at the 0.05 level.

** Significant effect at the 0.01 level.

*** Significant effect at the 0.001 level.

### Volatile Compounds

The percentages of the main volatile compounds are shown in Table 5. Linalool was the predominant compound, followed by eucalyptol, α-Bergamotene, eugenol, 1-Octen-3-ol, and β-cis-Ocimene. Except for eugenol, all volatile compounds detected were significantly affected by CV. “Eleonora” recorded the highest content of eucalyptol, α-Bergamotene, 1-Octen-3-ol, and β-cis-Ocimene, while “Italiano Classico” showed the highest value of linalool (Table 5). The biostimulant influenced the whole aroma profile with the highest content of linalool and eucalyptol obtained at B_0.30_ and B_0.15_ doses, respectively. The same compounds increased with increasing NSC (HF > HS) while the highest values of α-Bergamotene, eugenol, and β-cis-Ocimene were obtained using the HS solution. The CV × B interaction affected the entire profile of volatile compounds (Table 5). For “Eleonora,” the B_0.30_ dose increase linalool by 27.33%, compared to the control, in contrast to “Italiano Classico,” where the application of the biostimulant did not result in significant differences. Furthermore, for “Eleonora,” the B_0.15_ dose increased eucalyptol and 1-Octen-3-ol. The highest values of α-Bergamotene, eugenol, and β-cis-Ocimene were obtained from the Eleonora × Control combination. Relative to the B × NSC interaction, at both nutrient solution concentrations, the B_0.30_ dose increase linalool (+11.81%, on avg.) compared with Control. Regardless of dose, the biostimulant in the HS reduced eugenol and α-Bergamotene. The highest values of 1-Octen-3-ol (2.95%) were obtained from the combination of B_0.15_ × HS combination. Except for eucalyptol, all volatile compounds were affected by the CV × NSC interaction (Table 5). For “Eleonora,” the FS increased linalool and 1-Octen-3-ol, compared to the HS. The opposite trend was observed for α-Bergamotene, eugenol, and β-cis-Ocimene. For “Italiano Classico,” only linalool was affected by the different nutrient concentrations, with the highest values recorded by the Italiano Classico × FS combination.

**Table 5:** Analysis of variance and mean comparisons for volatile compounds in Eleonora and Italiano Classico genotypes grown hydroponically under two nutrient solution and dose of biostimulant.

**Treatment**	**1-Octen-3-ol**	**Eucaliptol**	**β-cis-Ocimene**	**Linalool**	**Eugenol**	**α-Bergamotene**
**Cultivar (CV)**						
Eleonora	2.73 ± 0.07	25.42 ± 0.63	2.24 ± 0.14	43.08 ± 1.23	3.86 ± 0.27	7.72 ± 0.55
Italiano Classico	2.38 ± 0.05	17.44 ± 0.30	1.67 ± 0.03	60.77 ± 0.30	3.72 ± 0.20	5.48 ± 0.15
**Biostimulant (B)**						
Control	2.48 ± 0.06b	20.78 ± 0.90b	2.11 ± 0.18a	49.33 ± 3.40c	4.35 ± 0.35a	7.39 ± 0.97a
B_0.15_	2.81 ± 0.10a	22.64 ± 1.83a	1.9 ± 0.16ab	51.3 ± 2.86b	3.28 ± 0.23b	6.27 ± 0.25b
B_0.30_	2.37 ± 0.05c	20.86 ± 1.06b	1.85 ± 0.10b	55.16 ± 1.93a	3.74 ± 0.17b	6.14 ± 0.15b
**Nutrient Solution Concentration (NSC)**						
Half Strength (HS)	2.53 ± 0.08	20.83 ± 1.06	2.13 ± 0.13	50.46 ± 2.49	4.09 ± 0.27	7.24 ± 0.63
Full Strength (FS)	2.57 ± 0.07	22.03 ± 1.09	1.78 ± 0.10	53.40 ± 2.09	3.49 ± 0.17	5.95 ± 0.18
**CV × B**						
Eleonora × Control	2.57 ± 0.1b	23.3 ± 0.74b	2.63 ± 0.16a	38.38 ± 1.6d	5.06 ± 0.46a	9.81 ± 1.31a
Eleonora × B_0.15_	3.10 ± 0.04a	28.59 ± 0.61a	2.06 ± 0.32b	42.00 ± 1.09c	3.010 ± 0.2c	6.91 ± 0.21b
Eleonora × B_0.30_	2.50 ± 0.02bc	24.37 ± 0.20b	2.03 ± 0.18b	48.87 ± 0.62b	3.50 ± 0.14bc	6.43 ± 0.21bc
Italiano Classico × Control	2.38 ± 0.03 cd	18.26 ± 0.68c	1.60 ± 0.06c	60.27 ± 0.63a	3.64 ± 0.36bc	4.97 ± 0.22d
Italiano Classico × B_0.15_	2.52 ± 0.11bc	16.69 ± 0.40c	1.74 ± 0.05bc	60.61 ± 0.47a	3.56 ± 0.41bc	5.62 ± 0.24 cd
Italiano Classico × B_0.30_	2.23 ± 0.07d	17.36 ± 0.18c	1.67 ± 0.05bc	61.45 ± 0.36a	3.97 ± 0.29b	5.84 ± 0.16 cd
**B × NSC**						
Control × HS	2.33 ± 0.02c	19.74 ± 0.98	2.18 ± 0.28	47.14 ± 5.43e	5.21 ± 0.40a	8.91 ± 1.71a
B_0.15_ × HS	2.95 ± 0.09a	22.14 ± 2.74	2.19 ± 0.26	49.82 ± 4.44d	3.04 ± 0.19c	6.30 ± 0.49b
B_0.30_ × HS	2.33 ± 0.06c	20.61 ± 1.58	2.01 ± 0.17	54.40 ± 2.95ab	4.03 ± 0.28b	6.51 ± 0.17b
Control × FS	2.62 ± 0.08b	21.83 ± 1.46	2.04 ± 0.24	51.51 ± 4.41 cd	3.5 ± 0.30bc	5.87 ± 0.50b
B_0.15_ × FS	2.68 ± 0.18b	23.14 ± 2.66	1.61 ± 0.11	52.78 ± 3.93bc	3.53 ± 0.43bc	6.23 ± 0.18b
B_0.30_ × FS	2.41 ± 0.09c	21.12 ± 1.57	1.68 ± 0.07	55.91 ± 2.73a	3.44 ± 0.13bc	5.76 ± 0.15b
**CV × NSC**						
Eleonora × HS	2.65 ± 0.12b	24.66 ± 1.00	2.62 ± 0.11a	40.94 ± 1.90d	4.34 ± 0.44a	8.95 ± 0.95a
Eleonora × FS	2.81 ± 0.08a	26.18 ± 0.74	1.86 ± 0.19b	45.23 ± 1.30c	3.38 ± 0.23b	6.48 ± 0.15b
Italiano Classico × HS	2.42 ± 0.09c	17.00 ± 0.35	1.64 ± 0.04b	59.98 ± 0.33b	3.85 ± 0.32ab	5.53 ± 0.22c
Italiano Classico × FS	2.34 ± 0.05c	17.88 ± 0.45	1.70 ± 0.05b	61.57 ± 0.32a	3.60 ± 0.25b	5.42 ± 0.21c
**Significance**						
**CV**	^***^	^***^	^***^	^***^	ns	^***^
**B**	^***^	^***^	^*^	^***^	^***^	^***^
**NSC**	ns	^**^	^***^	^***^	^***^	^***^
**CV× B**	^***^	^***^	^**^	^***^	^***^	^***^
**B × NSC**	^***^	ns	ns	^*^	^***^	^***^
**CV × NSC**	^***^	ns	^***^	^**^	^*^	^***^

Nutrient solution concentration treatments: HS, half strength; FS, full strength; Biostimulant treatments: Control; B_0.15_ = 0.15 ml L^−1^ of Trainer^®^; B_0.30_ = ml L^−1^ of Trainer^®^. All data are expressed as percentage relative abundance (%). ns, non-significant effect. Data represent means ± standard error of 3 replicates (*n* = 3). Treatment means within each column followed by different letters denote significant differences (*p* < 0.05) according to Tukey–Kramer HSD.

* Significant effect at the 0.05 level.

** Significant effect at the 0.01 level.

*** Significant effect at the 0.001 level.

### Principal Component Analysis

**Figure 2:**
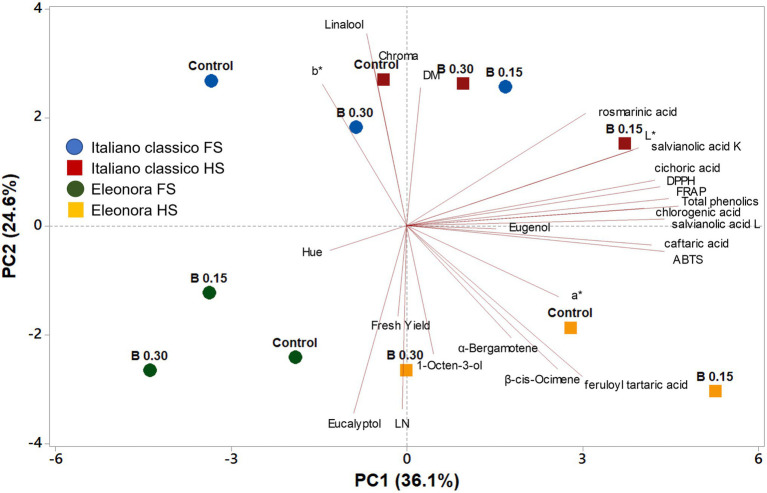
Loading plot and scores of principal component analysis (PCA) for yield, yield components, leaf colorimetric parameters, phenolic acids profile, antioxidant activities, and volatile compounds in two Genovese basil genotypes (Eleonora and Italiano Classico) grown hydroponically under two nutrient solution and dose of biostimulant [Nutrient solution concentration treatments: HS, half strength; FS, full strength; Biostimulant treatments: Control; B0.15 = 0.15 ml L^−1^ of Trainer^®^; B0.30 = ml L ^−1^ of Trainer^®^].

A principal component analysis (PCA) for yield, visual and quality attributes was conducted to further explore differences between the two “Genovese” basil genotypes (Eleonora and Italiano Classico), grown in a FRS with two different concentrations of NS (1 dS m^−1^-Half Strength [HS] and 2 dS m^−1^-Full Strength [FS]) and two doses of biostimulants (0.15 and 0.30 ml L^−1^, compared to an untreated control). The first two principal components (PCs) explained 60.7% of the cumulative variance, with PC1 and PC2 accounting for 36.1 and 24.6%, respectively (Figure 2). PC1 was positively correlated with all target polyphenols, volatile compounds as well as the antioxidant assays. Also, PC1 correlated negatively with the visual attributes (L, a^*^, b^*^). Furthermore, PC2 correlated positively with the three antioxidant activities and target polyphenols (Figure 2). Based on the angle between vectors of the examined variables, cichoric acid, chlorogenic acid, total phenols, DPPH and FRAP were found to be positively and significantly correlated among them (angle <90°) and negatively correlated with eucalyptol (angle >90°; Figure 2). The PC1 and PC2 score plot discriminated tested treatments into different cluster groups. On the positive side of PC1, “Italiano Classico” fertigated with HS and treated with 0.15 ml L^−1^ of PH delivered basil leaves of premium quality with high concentration of target polyphenols and antioxidant activities. At the lower right quadrant, “Eleonora” supplied with HS solution, showed the highest aroma profile, while the “Eleonora” cultivar fertigated with FS (irrespective of the biostimulant treatment) was positioned in the lower left quadrant distinguished by the poorest nutritional value (Figure 2).

## Discussion

### The PH’s in Nutrient Solution Boosted Basil Yield

Soilless systems are increasingly used to maximize the yields of premium quality vegetables. Among these, the FRS is characterized by ease of use, low management costs and high functionality, allowing early production with standard characteristics even on a large scale. The surprising yield obtained in the present study confirms the high efficiency of the FRS for basil cultivation compared to soil cultivation. Compared to the results obtained by Zheljazkov et al. (2008) on 38 basil cultivars grown in the open field, we recorded average yields approximately 15 times higher. This result is attributable to a better allocation of water and nutritional resources and the high density adopted (317 m^−2^; Ciriello et al., 2020). Regardless of the growing system, genetic material plays a crucial role in the productive response of basil (Žlabur et al., 2021). It should be noted that “Eleonora” showed better adaptability to the selected cropping system, producing more leaves per plant, and thus providing a higher fresh yield than “Italiano Classico,” which, in contrast, had a higher dry matter percentage (Table 1). In hydroponic systems, yield is primarily determined by the formulation of the NS, and to this end, numerous studies have focused on seeking optimal mineral levels to achieve *ad hoc* crop-specific “recipes” (Hosseini et al., 2021). For example, some studies have shown reduced yields in spinach (*Spinacia oleracea* L.; Cocetta et al., 2014) and lettuce (Hosseini et al., 2021) when grown in nutrient solutions with suboptimal mineral concentrations. Moreover, in our study, we did not observe any significant change in fresh yield in basil grown in HS (1 dS m^−1^) and FS (2 dS m^−1^) nutrient solutions. Our result is in line with the observations of Hosseini et al. (2021), who reported reductions in fresh yield in basil and lettuce grown on nutrient solutions with lower EC of 0.9 dS m^−1^ and corroborated the studies of Walters and Currey (2018) on “Sweet,” “Lemon,” and “Holy” basil, which did not observe yield increase with EC between 1 and 4 dS m^−1^. This shows that excess nutrients in the solution provide no benefit in terms of basil yield and negatively affect resource efficiency, economic viability, and environmental sustainability of hydroponic systems. The pressing need to ensure high yields of high-quality vegetables by adopting efficient and environmentally friendly cultivation methods makes the application of biostimulants in NS a promising ecological strategy. In our study, the application of PH’s (Trainer^®^) in the NS increased the fresh yield, the dry yield, and the number of leaves proportional to the dose used (Table 1). These results highlight that applying the biostimulant directly to the NS is a beneficial strategy to increase yield in hydroponic systems, as also shown by Cristofano et al. (2021) in lettuce. The beneficial effects of PH’s on yield parameters, also obtained in arugula (Caruso et al., 2019; Giordano et al., 2020), celery (*Apium graveolens* L.; Consentino et al., 2020) and basil (Rouphael et al., 2021), can be attributed to the peptides and bioactive amino acids characteristic of commercial formulations (Rouphael et al., 2021). Peptides, involved in cell differentiation and division, due to recognized hormone-like activity, modify root architecture and growth, improving uptake and crop yield (Li et al., 2016; Colla et al., 2017; Kim et al., 2019). The above effects are also attributable to amino acids (easily absorbed by roots) that are involved in essential signaling processes in addition to performing physiological functions (Tsouvaltzis et al., 2020). These molecules found in Trainer^®^ could have promoted nitrogen uptake in the rhizosphere and regulated key transcription factors and photosynthesis (Alfosea-Simón et al., 2021). In contrast to what was observed for the yield, the higher accumulation of dry matter in plants treated with the biostimulant (Table 1) is not widely supported in the literature. As an example, Caruso et al. (2019) recorded results comparable to ours in arugula, while Consentino et al. (2020) obtained opposite results in celery. In spinach, Rouphael et al. (2020) observed no significant difference for this parameter. The contrasting results highlight how the effects of biostimulants depend on factors such as time and mode of application, growth conditions, and genotype (Teklić et al., 2021). In line with the above, although the biostimulant, on average, increased the fresh and dry yield, integration of the PH’s into the FS and HS solutions showed dose-dependent responses. While, for the FS, increasing the dose led to linear increases in fresh and dry yields, there was no apparent dose-dependent effect for the HS. Under these operating conditions, the above points out that the impact of the biostimulant could also be influenced by the mutual interaction between the application dose and the concentration of the NS.

### Different Genotypes Impacted Visual Attributes of Basil Leaves

Color is a characteristic of light measurable in terms of wavelength and intensity, related to the observer’s perception and to the light conditions under which it is observed, able of influencing consumer choice about food quality (Pathare et al., 2013). In basil, the bright green color of the leaves and the attraction of consumer interest is a critical industrial requirement for the preparation of a “pesto” sauce, as it reduces the use of artificial colorants (Ciriello et al., 2021b). Although the basil genotypes tested all belonged to the “Genovese” cultivar., CIELab colorimetric parameters (L, a^*^, b^*^) showed cultivar-dependent variations, with “Italiano Classico” recording the highest values of all above parameters, confirming the results of Ciriello et al. (2020) on the same basil genotypes grown in FRS. The higher values of L (brightness) and a^*^ (greenness) in “Italiano Classico” are consistent with Chroma values, indicating a higher color intensity perceived by the consumer. The latter parameters (L and a^*^) were also influenced by the NSC. In particular, the FS increased leaf brightness (higher L) and greenness (higher a^*^) compared to the HS, although it did not show any productive differences (Table 2). As argued by Fallovo et al. (2009), the more intense green leaf color could be attributed to a higher chlorophyll content (data not shown) related to the higher nitrogen levels of the FS (2 dS m^−1^). Our color results showed that applying the biostimulant at the highest dose (B_0.30_) in the NS increased only the parameter a ^*^, compared to the Control, in agreement with Consentino et al. (2020). This result could be related again to the increase induced by biostimulants in chlorophyll content, as observed by Vernieri et al. (2006) and Aktsoglou et al. (2021) in arugula and peppermint (*Mentha × piperita*), respectively. However, Caruso et al. (2019) and Giordano et al. (2020), despite observing an increase in SPAD (an indirect index of chlorophyll content), did not record a change in color in arugula after the application of PH’s. These results confirm once again how the effects of biostimulants differ primarily by species, but also by dose, mode of application, and different growth and development conditions.

### Impact of Interactions Between Investigated Factors on Basil Quality Attributes

The inability to “escape” from possible environmental threats has “bound” plants to passive defense mechanisms based on the production of specialized metabolites that have allowed their survival over time (Trivellini et al., 2016). In medicinal plants, specialized metabolites are characterized by significant structural and chemical diversity that uniquely confers the desired technological and nutritional attributes (Dias et al., 2016; Filip, 2017). Although we had used “Genovese” genotypes characterized by a similar phenolic profile in our study, the concentration of total phenolic acids differed considerably (Table 3). The higher total phenolic concentration in “Italiano Classico” (Table 3), also obtained in other works conducted under different growth conditions, again demonstrates how the accumulation of these compounds is strongly influenced by genetics (Žlabur et al., 2021). Despite this, the antioxidant activities reported in Table 4 were not affected by the effect of the cultivar. The explanation for this could lie in the fact that between the two genotypes tested there was only a 9.4% difference in the concentration of total phenols, but it could also be due to the synergistic effects between polyphenols and other chemical constituents, such as ascorbic acid and carotenoids that contribute to overall antioxidant activity (Graziani et al., 2021). The data in Table 3 clearly show the influence of genetics on the diversity of the phenolic profile of the basil genotypes. Although rosmarinic acid is referred to as the most represented phenolic acid in basil (Kiferle et al., 2013; Filip, 2017; Ciriello et al., 2020), in our study, both “Eleonora” and “Italiano Classico” were characterized by a predominant concentration of chicoric acid. The influence of genotype on the predominant biosynthesis of chicoric acid was also confirmed by Kwee and Niemeyer (2011) in basil. The authors showed that 9 basil varieties out of 15 tested had the highest absolute concentration of chicoric acid. Furthermore, it is important to note that the discrepancy with the results reported in the literature is attributable not only to the genetic material but also to the different extraction methods and solvents used to determine the phenolic acids and the different growth conditions adopted (Filip, 2017). Regardless of the cultivar., the present work confirms that basil leaves contain, in addition to high levels of chicoric acid, significant amounts of salviolanic acids K and L. The important and recognized pharmacological properties of salviolanic acid could further increase the nutraceutical value of basil (Prinsi et al., 2020). The change in the entire phenolic profile in response to changing concentrations of NS (Table 3) confirms that nutritional stress can affect the biosynthesis and accumulation of specialized metabolites (Mahajan et al., 2020). The use of a HS increased the levels of the entire phenolic profile in both genotypes compared to what was observed in the FS, similar to what was observed in basil (Jakovljević et al., 2019), lettuce (El-Nakhel et al., 2019), artichoke (*Cynara cardunculus* subsp. *scolymus* L.), and cardoon (*Cynara cardunculus* L.; Rouphael et al., 2012). The increase in the phenolic profile showed the same trend as the ABTS, DPPH, and FRAP assays (Table 4), indicating how the limitation of nutrition induced an improvement in antioxidant activity. This result and the increase in the phenolic profile are probably related to the halving of nitrate in the HS, which as observed by Chishaki and Horiguchi (1997) has a more significant influence on the accumulation of phenolic acids than potassium and phosphorus deficiency. Low nitrogen levels would stimulate phenylpropanoid metabolism, inducing the accumulation of phenylalanine ammonia-lyase (PAL) and other critical enzymes involved in the biosynthesis of phenolic compounds (Fritz et al., 2006; Wada et al., 2014; Mahajan et al., 2020). This would suggest that low nitrogen levels, by decreasing growth requirements, would promote the accumulation of specialized metabolites (Prinsi et al., 2020). However, in our study, we did not observe a reduction in fresh yield at HS (Table 1), which justifies the high phenolic concentration as a result of ex novo synthesis rather than a deceleration of primary metabolism by increased activity of PAL or its substrate (phenylalanine; Gershenzon, 1984). Similarly to the yield parameters (Table 1), the application of the biostimulant in the NS significantly increase the phenolic concentration in basil. A probable reason for elucidating this interesting result could be related to the increase in production due to a better photosynthetic activity mediated by the biostimulant, which would have promoted secondary metabolism (Colla et al., 2017). However, the bioactive signal molecules characteristic of PH’s, in addition to providing the plethora of physiological effects mentioned above, may have triggered the induction of the production of specialized metabolites. Based on a recent work (Kisa et al., 2021), in which a positive influence was observed on basil secondary metabolism after applying amino acids, our results could be traced to the composition of Trainer^®^, which is characterized by the presence of these organic molecules. One of the crucial functions of amino acids and molecules derived from them is their ability to serve as precursors for specialized plant metabolites, acting both as substrates and as activators of key enzymes such as chorismate mutase, creating points of interconnection in the biosynthesis of phenolic compounds (Feduraev et al., 2020; Kisa et al., 2021). Interestingly, regardless of the cultivar and concentration of the nutrient solution, among the biostimulant doses tested (B_0.15_ and B_0.30_), the highest accumulation of total phenolics was obtained after application in the nutrient solution of the lowest dose (B_0.15_) of the biostimulant, confirming that this result was not dose dependent. The justification behind the above could stem from the fact that at dose B_0.30_, the biostimulant prioritized production over secondary metabolism. The variability in the composition of essential oils among basil types gives this aromatic herb a multitude of uses. The non-unique aroma of basil is determined by the various compounds that constitute its essential oils, mainly terpenoids (synthesized through the mevalonate pathway and the 2-methylitritol 4-phosphate pathway) and phenylpropanoids (synthesized through the shikimate pathway; Dudai et al., 2020; Mahajan et al., 2020). The distinctive aroma of “Genovese” basil and its derivative products (such as pesto sauce) is attributable to the dominant presence of critical aromatic molecules such as linalool and the complete absence of mint (menthol) and anise (estragole; Ciriello et al., 2021b). Not surprisingly, in the basil genotypes tested, linalool was the predominant, a compound that, in addition to uniquely characterizing the flavor of the “Genovese” genotypes, also has documented therapeutic properties (Mughal, 2019). However, the differences found in “Eleonora” (higher content of 1-Octen-3-ol, eucalyptol, β-cis-Ocimene, and α-Bergamotene) and “Italiano Classico” (higher content of linalool) in the full aroma profile reported in Table 5 underscore the significant impact of genotype. These differences could be due to the different leaf morphology, the density of oil glands, vegetative growth, and biosynthesis of volatile odorous compounds (Khammar et al., 2021). Compared to the latter, the higher content of linalool but lower contents of eucalyptol and β-cis-ocimene contents, recorded in “Italiano Classico,” compared to “Eleonora,” highlights a clear genotypic effect on gene expression that regulates the conversion of its sole precursor (geranyl pyrophosphate) from the enzymes linalool synthase, 1,8-cineole synthase and β-cis-ocimene synthase (Chang et al., 2007). The basil genotypes tested showed a different response to the biostimulant (Table 5). As seen in peppermint and spearmint (*Mentha romana* L.; Aktsoglou et al., 2021), biostimulant in the NS did not result in any significant difference in the composition of the aroma profile of Italiano Classico. On the one hand, this result could indicate a low sensitivity of the cultivar to the biostimulant and, on the other hand, it could result from the use of insufficient doses to induce alterations in the overall composition of volatile oils. On the contrary, in “Eleonora,” there was a significant effect on the whole aromatic profile caused by the application of the biostimulant. We observed a direct correlation between increasing the dose of biostimulant and the linalool content, contrary to what was observed for eugenol, β-cis-Ocimene and α-Bergamotene, which instead decreased regardless of the dose used. Since plant nutrition is known to influence the content of volatile oils (Aktsoglou et al., 2021), it is not surprising that the use of NS at different concentrations resulted in significant differences in basil flavor profile (Table 5). As with the biostimulant, different responses were observed for the NSC between the two basil genotypes used in the present study. In “Italiano Classico,” the different NSC changed only the content of the most represented compound (linalool), while in “Eleonora,” all compounds, except eucalyptol, were significantly affected by the different availability of nutrients in the nutrient solution. In any case, in both genotypes, the more concentrated nutrient solution (FS) increased the linalool content. As also seen on *Salvia sclarea* L. (Sharma and Kumar, 2012), the higher availability of nutrients, especially nitrogen, led to an increase in the linalool content, as nitrogen, involved in the biosynthesis of primary and secondary metabolites, could positively interact in its metabolic pathway, confirming our results (Khammar et al., 2021).

## Conclusion

The challenge imposed on the agricultural sector to provide nourishment to a growing population has led to alternative production techniques such as hydroponics. However, the urgent need to reduce chemical inputs in alternative cropping systems has paved the way for biostimulants, which currently represent an environmentally sustainable strategy for horticultural production. Under the experimental conditions of our study, the varietal comparison showed that “Eleonora” provided the highest fresh yield (6576.81 g m^−2^). At the same time, “Italiano Classico” had the highest total phenol concentration (1590.04 μg g^−1^ dw). The use of NS with different concentrations did not result in significant differences in fresh yield, regardless of the cultivar., but positively impacted the aroma and phenolic profile. Specifically, the HS increased total phenols by 32.5%, compared to the FS that ensured the highest content of eucalyptol (22.0%) and linalool (53.4%). The application of biostimulants in the NS increased all biometric parameters (such as the number of leaves, fresh and dry yield) and the linalool content proportionally to the dose used, while the highest total phenol concentration was obtained from the lowest dose (B_0.15_). Based on the excellent results achieved, the application of biostimulants in NS turned out to be a valid strategy to reduce chemical input. For this reason, it should also be investigated on other leafy crops to define a new production technique that can improve both yield and quality.

## Data Availability Statement

The raw data supporting the conclusions of this article will be made available by the authors, without undue reservation.

## Author Contributions

MC, SP, and YR: conceptualization and project administration. MC, LF, MK, GC, GG, and AR: methodology, validation, formal analysis, investigation, and writing—original draft preparation. MC and LF: software, resources, and data curation. MC, LF, and YR: writing—review and editing. SP and YR: visualization, supervision, and funding acquisition. All authors contributed to the article and approved the submitted version.

## Funding

This research was conducted in the framework of a PhD project sponsored by the Italian Ministry of Education (PON research and innovation).

## Conflict of Interest

The authors declare that the research was conducted in the absence of any commercial or financial relationships that could be construed as a potential conflict of interest.

## Publisher’s Note

All claims expressed in this article are solely those of the authors and do not necessarily represent those of their affiliated organizations, or those of the publisher, the editors and the reviewers. Any product that may be evaluated in this article, or claim that may be made by its manufacturer, is not guaranteed or endorsed by the publisher.
